# Development and perspectives of theological bioethics

**DOI:** 10.3325/cmj.2013.54.86

**Published:** 2013-02

**Authors:** Luka Tomašević

**Affiliations:** Faculty of Catholic Theology, University of Split, Split, Croatia

The beginning of the 21st century is marked by a *great revolution* supported by science, which is called *biothechnological revolution* and our century a *biotechnological* century (1). Enormous advances in biology, especially in genetics have led to homological and heterological procreation in laboratory, human genome manipulation, genetic engineering, animal but also human cloning, and scientific research on human embryos for therapeutic or eugenic purposes.

Philosophy of life has changed: people no longer recognize the value of life, they rather talk about the quality of living. This point of transition from *holiness of life* to its quality had an impact on the quality of human relationships. Furthermore, another consequence is that life is no longer perceived as being exclusively in God's hands, but in our hands as well. Therefore, it can be said that the present comprehension of life is rather *subjective*.

From the point of view of Christianity, life is a precious gift from God. This gift has to be developed and preserved by people, who have never been masters of life but rather its servants. Consequently, any Christian-theological view of life is quite *life-giving*. This article presents the development and basic principles of Catholic and Orthodox bioethics.

## Theological bioethics

The emergence of bioethics is linked to V. R. Potter II, professor at the Wisconsin University, USA, who named it “the science for survival.” A generally accepted definition of bioethics is that it is a “systematic research of human behavior at the scientific and health care fields as long as this behavior is analyzed in the light of moral values and principles.” The term bioethics today implies human responsibility for all the forms of life that exist in the world (so called biocentrism) (2). Theological bioethics (3) can be defined as follows: “Bioethics is a part of moral philosophy dealing with permissibility or impermissibility of interventions or manipulations with human life, especially related to the practice and the progress of medical and biological science.” (4)

Bioethics was generated in Christian cultural context and it is easy to observe the close historical connection between medical ethics and Christian tradition and principles. Although theological bioethics is not supported by secular bioethics because of its social-conservative orientation, from the very beginning religious activists have been taking part in the struggle for political and human rights and raising the awareness of religious and human values in the society. Theologians, especially those belonging to the Catholic Church, began to talk about the *common good* as a traditional social truth of Catholic social doctrine and to demand justice in the area of integrated life and health insurance. In this context, Catholics initiated the discussion about embryo’s and fetus’s right to live, which gave rise to Pro Vita Catholic movement.

According to the Bible and Christian tradition, the principal values of any human activity should always be man and life. Man is not any being but rather human being that God created for Himself, in His own image, as His interlocutor and sucreator (5). Though created as a dependent and limited being, man has his immense dignity. In the Bible, man perceives himself as an active, creative, and responsible being. He is not an absolute master, neither of himself nor of the worldly life but merely a responsible manager. So, God created the world and man, and left all that to man's governance. It is interesting that God did not give man any *working* program, but rather reason and freedom to discover and follow the laws of life. Man is therefore an integrated part of nature.

The Christianity has also introduced into bioethics one of its specific concepts – the concept of *love*. Love is the source of moral Christian life and the essence of the very Christian proclamation of Christ. Actually, God was the first to love us freely, so we are to love each other (1 John 4, 10), and the culmination of such love was shown by Christ Himself when He laid down his life for others (John 15, 13). Therefore, for any Christian He is real the “measure” of love. According to such law of love, Christians have to recognize each other (John 13, 35) and deeds of love should be their truly Christian preoccupation. Thus, Christian love is taken as a form of all virtues.

There's also another great concept of Christian tradition, the notion of *justice*, which is part of the discussion about *health care* and its equitable distribution. Accordingly, human health becomes a global right of every human being. Christian justice emphasizes that all people are equal whether they are rich or poor, and that they have an equal right to treatment. Therefore, Christianity highlights that the religious and moral values do not belong to any political orientation, right or left, and that poverty can become a worldwide common field of action for conservatives as well as liberals (6).

## The emergence of Christian Catholic bioethics

Western or Catholic Christianity developed the so called *metaethical* reflection, which properly differentiates the value of life from any other anthropological concept. The Catholic Church elaborates the relationship between personal life and sexuality within the framework of axiological science, but it also encompasses embryo-political and technical science issues as they include possible manipulation with the sources of life. Obviously, such bioethics has not merely preserved its traditional deontological Catholic views but also searched for new ways of “bioethical interference” in religion, science, and humanistic culture. Its “bioethical interference” was nothing more than Potter's “bridge.” Especially important for *Catholic Bioethics* is the papal encyclical *Evangelium vitae* (7). *Evangelium vitae* is the eleventh encyclical of Pope John Paul II., completely dedicated to the burning questions of new medical ethics (7). Life is the hermeneutic key of Catholic bioethics. It is directed both by man and God. Thus, it is both man's history and God's mission.

Another important issue in Catholic bioethics is *freedom,* which is the base of man’s dignity. “God gives freedom to each individual. Freedom as a such includes *essential relational dimension*. It is a great gift from God, the Creator, put in the service of a person and his/her accomplishment through self-giving and accepting of others.” (8) Such freedom is justified through each individual having his/her personal orientation and responsibility to his/her mission.

Catholic bioethics implies *Agape structure* of love, ie, receiving and giving love according to which medicine is a *mission* rather than a profession, and patients are physicians’ brothers. Only through received and given love, individuals are able to come closer to each other and to create dimensions of the meaning of life and illness. It is disinterested love that is given and transformed in *love serving*. Such love would never cause any discrimination among patients, but would rather care for whole life and life of all.

In conclusion: our biological nature must not be observed apart from the sense it has for us. It implies that man, due to his reason, has to find the sense of his structures but also to accept and integrate his biological nature for the sake of humanity. Otherwise, biological process itself does not create moral demand, it is rather created by its attachment to human person, ie, belonging to interpersonal relationship and having the dignity of an agent in God's plan and the possibility of bearing future life.

## Emergence and development of Orthodox bioethics

Orthodox bioethics bases its ethical judgments differently. Orthodox Christianity believes that it is the true source of Christ’s Church and that it has to serve to its people as a spiritual and moral directing post through life (the soteriological mission). Orthodoxy points to the fact that people are given guidelines by God and these guidelines should not be taken arbitrarily or easily. They have to reflect the faith confessed by the Church and should be based on the fundamental truths of the Orthodox teaching, which as the proclamation of God is found in Christ’s Church. Based on such reflections, Orthodox ethics gives fundamental guidelines and answers to contemporary questions and issues referring to bioethics (9).

However, Orthodox ethics has only few normative regulations. Its ethical judgments are based on the Holy Scripture and Holy Tradition. The holy tradition consists of the “*mind of the Church*” and is discerned in the decisions of ecumenical and local councils, the writings of the Holy Fathers of the Church, canon law, and the penitentials. Modern issues are elaborated in accordance with the “mind of the Church,” and any doctrine is subjected to episcopal, synodical, or general Church criticism.

Orthodox ethicists maintain that new ethical guidelines should be based on the parallels in the tradition (10). Orthodox theological anthropology is based on the fact of human *likeness to God* as a given and a potential. Some of the patristic authorities distinguish between the creation of human beings in God’s “*image” *and in God’s “*likeness*.” “*Image*” is the *donatum* of intellect, emotion, ethical judgment, and self-determination. In fallen humanity these remain part of human nature, albeit darkened, wounded, and weakened. The “*likeness*” is the human *potential* to become *like God*, ie, Godlike, to achieve an ever expanding, never completed perfection. This fulfillment of our humanity is traditionally referred to as *theosis* or *divinization*. To achieve *theosis* means to realize our full human potential. Ethically, this teaching leads to the acceptance of the existence of *human nature*, but it clearly does not restrict our *humanum* to conformity to that nature. The “*image*” provides a firm foundation for ethical reasoning. The “*likeness*” offers the basis for ethical reasoning.

Orthodox Christianity teaches that, though God is the Lord of history, he does not coerce or force obedience and conformity to his will. Forced conformity would be *dehumanization*, whereas fulfilled humanity – which is the divinization of human life – must be free, since God is free. This raises the question of Divine Providence and Human Responsibility.

Moreover, the principle of love not only separates ethics from the legal system but also presents a light-motif of any activity. In this sense, love is very important for any action as the Holy Trinity is united in love, so the Church teaches that being Godlike means being filled with love. According to this, any love-based action is in harmony with all that God gave us in his Commandments.

The first efforts aimed at providing a comprehensive Orthodox ethical teaching on bioethical questions were presented in the first edition of the *Encyclopedia of Bioethics* (1978), by Samuel Stanley Harakas, professor of moral theology at the Holy Cross Theological School, Boston (11). Until its publication, Orthodoxy did not discuss bioethical issues, whereas the problems of life, health, death, and birth were treated according to the documents of special moral theology, God’s Commandments, or Christian duties.

The work of Harakas was the only one dealing with Orthodox ethics for twenty years, until the publication of the first manual of Orthodox bioethics (12) by John Breck, S. Vladimir’s seminary professor (New York) in 1998. Two years later, in 2000, Tristram Engelhardt, converted to Orthodoxy and published a manual entitled the *Foundations of Christian Bioethics* (13).

As opposed to East European Orthodox Church, West European Orthodox Church was not particularly interested in bioethics, and regarded bioethics as the last Western heresy. However, it condemned genetic manipulation. The Greek Orthodox Church, on the other hand, has its own *Bioethical Commission* within its Holy Synod and bioethical issues have been discussed several times by Christodoulos, the archbishop of Athens. Bioethical writings were also present in Orthodox churches under Communism. At its Holy Synod held in 2000 (Moscow), the Russian Orthodox Church made a regulation titled *The Basic Social Concept of the Russian Orthodox Church*, which deals with the concept of bioethics and its issues in the chapter 12, and which actually inspired the writing of this article (11).

In conclusion, we can say that the Catholic Church has willingly faced bioethical challenges, first in the USA and then in the rest of the world, with its clear attitudes inspired by the Gospel. The development of bioethics in the Orthodox Church has been somewhat slower, beginning in the USA and spreading throughout western European, and after the fall of communism, eastern European countries.
